# Ethical Issues in Living Donor Kidney Transplantation: An Update from a Psychosocial Perspective

**DOI:** 10.3390/healthcare12181832

**Published:** 2024-09-13

**Authors:** Valentina Martinelli, Estella L. L. Lumer, Matteo Chiappedi, Pierluigi Politi, Marilena Gregorini, Teresa Rampino, Andrea Peri, Andrea Pietrabissa, Laura Fusar-Poli

**Affiliations:** 1 General Surgery Unit 2, IRCCS Policlinico San Matteo, 27100 Pavia, Italy; a.peri@smatteo.pv.it (A.P.); a.pietrabissa@smatteo.pv.it (A.P.); 2 Harvey Medical Course, Department of Molecular Medicine, University of Pavia, 27100 Pavia, Italy; estellalindalu.lumer01@universitadipavia.it; 3 Child Neurology and Psychiatry Unit, ASST Pavia, 27029 Vigevano, Italy; matteo_chiappedi@asst-pavia.it; 4 Department of Brain and Behavioral Sciences, University of Pavia, 27100 Pavia, Italy; pierluigi.politi@unipv.it (P.P.); laura.fusarpoli@unipv.it (L.F.-P.); 5 Nephrology, Dialysis and Transplant Unit, IRCCS Policlinico San Matteo, 27100 Pavia, Italy; m.gregorini@smatteo.pv.it (M.G.); t.rampino@smatteo.pv.it (T.R.); 6 Department of Internal Medicine and Therapeutics, University of Pavia, 27100 Pavia, Italy; 7 Department of Clinical-Surgical, Diagnostic, and Pediatric Sciences, University of Pavia, 27100 Pavia, Italy

**Keywords:** ethics, living donor kidney transplantation, psychosocial outcomes, autonomy

## Abstract

Living donor kidney transplantation (LDKT) currently represents the treatment of choice for patients with end-stage renal failure. LDKT is a serious event with profound psychological, interpersonal, familial, and social implications. Over the last few years, there has been an exponential growth in living donation programs involving genetically and emotionally related donors, as well as people who donate to an unrelated and unknown subject. The implementation of paired exchange programs, Samaritan donation, and preemptive transplantation raise further ethical issues, which are inextricably linked to the unique psychosocial context of both the donor and the recipient. The present narrative review aims to provide an update on the main ethical challenges related to LDKT. We conducted a comprehensive literature search in PubMed/Medline. The results of the most relevant studies were narratively synthesized from a psychosocial perspective around the four principles of biomedical ethics: autonomy, beneficence, non-maleficence, and justice. Finally, we discussed the potential future directions to provide an effective, patient-centered, and ethical psychosocial assessment and follow-up of living donors and recipients that underwent LDKT.

## 1. Introduction

The global prevalence of chronic kidney disease (CKD) is estimated to be around 10–15%, with variations depending on the countries, risk factors, and definitions used. CDK will likely become the fifth most common chronic condition by 2040 [1]. Kidney transplant recipients have a longer life expectancy and a better quality of life than patients receiving dialysis [2]. However, despite a global increase in the number of kidney transplants across Europe, the shortage of organs available for donation still represents a major challenge in transplantation [3].

Although living donor kidney transplantation (LDKT) currently represents the primary treatment option for patients with end-stage kidney disease (ESKD), the number of LDKT performed each year is still limited, with significant disparities among countries. This gap may be related to high rates of organ disqualification, heterogeneity in healthcare systems organization and resources, as well as different attitudes to living donation, which are partially influenced by the social context [4,5]. To date, 5833 patients in Italy are on the waiting list for a kidney transplant, with an average waitlist time of 3.2 years [6]. Out of the 2245 kidney transplants performed in 2023, 335 (15%) were LKDT [7].

On the one hand, evidence has shown that LDKT recipients have superior outcomes compared to those who undergo other available treatments due to medical and technical aspects (e.g., a reduction in the waiting time and an avoidance of dialysis in the case of preemptive transplantation, the possibility of planning surgical operations, and the careful selection of donor candidates) [8]. On the other hand, LDKT is a complex procedure that implies many psychological and ethical issues, which are mostly derived from the human source of the graft. Even today, 70 years after Joseph Murray performed the first successful kidney transplantation in humans between two identical twins at Brigham and Women’s Hospital (Boston), LDKT raises an intense debate. Murray himself, in his Nobel lecture, commented the following: “*The only remaining problem was the ethical decision concerning the removal of a healthy organ from a normal person for the benefit of someone else. For the first time in medical history, a normal healthy person was to be subjected to a major surgical operation not for his own benefit*” [9].

Nephrology and transplantation medicine have been deeply interwoven with the birth of bioethics. In 1979, Childress and Beauchamps introduced the four key principles of biomedical ethics—i.e., autonomy, beneficence, non-maleficence, and justice—which still represent the cornerstones of ethics in healthcare practice [10]. These principles provide a conceptual framework to reflect on clinical material and reach reasoned conclusions on controversial matters. LDKT represents a paradigmatic example of a potential conflict between a duty of beneficence toward the transplant recipient and a duty of non-maleficence toward the donor, making the assessment of a living donor an ethically fraught issue [11].

The debate on operating on an individual who does not need and is put at a quantifiable risk from a surgical procedure, with potential effects on their quality of life, is still ongoing [12]. The potential risks imposed by the process on the donor cannot be ignored. Nevertheless, technical advances have allowed surgeons to utilize minimally invasive surgical techniques on the donor, reducing risks and leading to a positive benefit/risk ratio when the donor-recipient dyad is considered [11,13].

Whether living kidney donation creates a tolerable risk or not represents a major issue, although it is still difficult to find a clear answer. Even in the presence of all cautions being undertaken in the evaluation and follow-up phases, the possibility of future unexpected traumatic events—e.g., a donor’s trauma involving their only remaining kidney—cannot be excluded. While in all complex and multifaceted situations, the person involved is the only one who can actually decide, in the case of LDKT, one should obtain consent from both members of the dyad (i.e., the donor and the recipient). Specific attention to the donor with detailed, methodical, and objective clinical and instrumental evaluations should be shared and discussed with the transplant team. International guidelines and recommendations clearly state that, regardless of potential recipient benefit, the safety and welfare of the potential living donor must always take precedence [14].

To maximize benefits and ensure the donor’s safety, living donation programs require a thorough medical screening of donor candidates within the framework of international guidelines and following specific standards [14,15]. Good clinical practice requires donors to be in almost perfect health to guarantee the safety of the surgical procedure and minimize their likelihood of experiencing harm following the donation. At present, a careful psychosocial evaluation of candidate donors is also strongly recommended to assess their psychological status, emotional stability, motivation to donate, as well as the quality of the relationship with the recipient [16].

The continuous advances in surgical techniques and immunological research have resulted in new options but have raised further ethical issues. These days, LDKT programs have been expanded to involve related living donors, non-related living donors, paired kidney exchange programs, and altruistic donations [17]. Although prior research has reported that living donors usually experience psychological and emotional benefits and improved perceived health and quality of life just after the intervention [18,19], recent data have revealed a more nuanced perspective during a longer follow-up period when psychosocial aspects were assessed more in-depth [20,21].

In the past decade, psychosocial and socioeconomic issues have emerged as important outcomes of LDKT. Psychosocial aspects cover a broad range of emotional, psychological, psychopathological, social, and financial variables. The most commonly investigated psychosocial aspects in kidney transplantation are health-related quality of life and psychological distress, including symptoms of depression and anxiety, fatigue and pain, and socioeconomic status. Expert recommendations have highlighted the need to explore these issues in LDKT, especially in the contexts of kidney exchange programs and anonymous donation [22,23].

Moreover, in recent years, transplantation programs, including LDKT, have faced new challenges, due to the COVID-19 pandemic, with regard to safety, allocation, and the availability of scarce medical resources and vaccination policies [24]. In March 2024, the first xenotransplant of a pig’s kidney in a 62-year-old man living with ESKD was conducted in the United States. The organ was genetically edited to prevent rejection and infections. The Food and Drug Administration (FDA) currently approves xenotransplants for compassionate use only [25]. This novel procedure, greeted as a major milestone in the effort to provide available organs to patients in the future, raises additional clinical and ethical issues as it offers a potentially viable alternative to LDKT.

The aim of this narrative review is to discuss the main current ethical challenges in performing LDKT for both recipients and donors and to inform clinical professionals in the field. Ethical topics will be explored through the four key principles of biomedical ethics: autonomy, beneficence, non-maleficence, and justice.

## 2. Materials and Methods

A search of the literature was conducted on PubMed and Medline in order to identify articles that explored the ethical issues related to kidney transplantations, both in relation to donors and recipients. Studies were retrieved using the following keywords: (ethics OR “ethical issues” OR autonomy OR beneficence OR “non maleficence” OR justice OR “psychosocial evaluation” OR “psychosocial outcomes”) AND (“living donor kidney transplantation”).

The search was limited to papers published from January 2004 to June 2024 written in English, Italian, and French. Two authors (V.M. and E.L.L.L.) independently screened articles by title and abstract to identify the most relevant publications (original papers and reviews) addressing this topic. The selected papers were critically revised, and any disagreement was solved through discussion with a third author (M.C.) to reach a consensus. All the co-authors reviewed and discussed the resulting draft to provide a theoretical point of view organized around the four key principles of biomedical ethics. The final version of the manuscript was then recirculated and approved by all the co-authors.

The theoretical model was designed to be conformant with Italian regulations on this topic. In Italy, living kidney donation is, in fact, strictly regulated by the law (Legge 26 June 1967 n.458) and specific protocols (Conferenza Stato-Regioni, 31 January 2002). Living donation is permitted between genetically (first-degree relatives) and/or emotionally related (e.g., partners and friends) pairs and to the benefit of an unknown person in the absence of any kind of familial or emotional relationship (i.e., altruistic or Samaritan donation). According to legislation, this type of donation must be free, autonomous, and aware. The final ascertainment of the donor’s clinical situation and motivations is under the jurisdiction of a third-party commission [15].

## 3. Results

### 3.1. Autonomy

Autonomy refers to an individual’s right to self-determination and to their ability to make decisions about medical treatments based on their own beliefs and values. The autonomy of an individual is at the foundation of their ability to express informed consent to medical and surgical procedures. Autonomy implies three main aspects: intentionality (i.e., the willingness to undergo the specific procedure); understanding (i.e., the ability to understand what will happen, in terms of the procedure and of its expected, possible, or probable consequences, both beneficial and harmful); and non-control (i.e., the absence of undue influence or coercion) [10,26]. All these aspects deserve careful attention and assessment in LDKT.

Specifically, LDKT involves two subjects, the donor and the recipient, whose decision-making, health, and quality of life are both independent and interdependent. An ethically sound approach to living donor transplantation must include a comprehension of the psychosocial dynamics involved in kinship and relational matters [27].

The assessment of autonomy is one of the core aims of the psychosocial evaluation in LDKT. To date, many psychosocial screening guidelines and protocols have been produced for kidney donors. According to guidelines, the donor has to be “competent, willing to donate, free from coercion, medically and psychosocially suitable, fully informed of the risks, benefits and alternative treatments available to the recipient” [28]. Although there is a general consensus on the content of the psychosocial assessment, this process still remains heterogeneous among different countries and transplant centers, concerning the professionals involved, the placement within the entire evaluation process, and the amount of time devoted. The key domains assessed by the psychosocial evaluation in LDKT have been clustered into six groups: motivation and decision-making; personal resources; psychopathology; social resources; ethical and legal factors; and information and risk processing [29]. Only recently have researchers developed standardized tools for the psychosocial assessments of kidney donors, including the Stanford Integrated Psychosocial Assessment for Transplant (SIPAT) in the United States [30] and the ELPAT living organ donor Psychosocial Assessment Tool (EPAT) in Europe [27].

The basis for autonomy and consent and the prerequisite for understanding is to provide the patient with adequate and substantial information about the procedures. Over several decades, advances in surgical techniques and immunological research have led to new options involving subjects who would have been excluded from LDKT in the past. One notable and widespread example regards paired exchange programs, which was first developed in the 1980s and subsequently expanded on to include cross-over transplantation and kidney transplant chains started by a Samaritan donation. These procedures may imply complex notions and require in-depth appropriate information tailored to a donor’s unique needs; therefore, it is crucial to consider the donor’s cognitive level and general knowledge to be fully illustrated and, in turn, understood. True informed consent is a process that requires time [13]. However, time is a major critical issue in clinical practice, and the psychosocial evaluation process may vary in terms of duration and placement within the transplant evaluation trajectory. Medical urgency and specific requirements, as in preemptive transplantation, may accelerate the entire process. The pre-donation period is often a stressful experience for the donor [20]. According to a recent review on the psychological impact of living kidney donation, some donors have described the pre-donation phase as a very anxious and uncertain period, in which they were confronted with their own dilemmas, the fear of being excluded for medical reasons, and the long wait time for results. In this context, it has been reported that some donors may use strategies to influence the decision of the transplant team by minimizing the descriptions of their personal fragility, previous psychological difficulties, or even withholding some information [20].

A qualitative study by Halverson et al. involving 20 individuals who donated a kidney to their first-degree relatives reported that, although participants described their decision to donate as obvious and free from external pressures, they indirectly expressed some ambivalence, especially regarding the rapidity of the process and their concern about exposing an intimate to the risks of living donation [27]. From a broader family perspective, McKinney et al. reported that the families of patients waiting to be transplanted suffered greatly in many aspects of their social lives and had difficulties in making informed decisions [31].

Psychological defense mechanisms activated by recipients and donors to face the emotional distress derived from the transplantation experience may also have an impact on understanding and decision-making. Defense mechanisms may be defined as the “tendentially involuntary feelings, thoughts or behaviors, which arise in response to perceptions of danger to the subject and are aimed, more or less adaptively, at hiding or alleviating the conflicts or stressful agents they give origin to anxiety or anguish” [32].

De Pasquale et al. used the Defense Style Questionnaires [32] to investigate the defense mechanisms of 50 transplant recipients, 11% of whom received a kidney from a living donor. Study participants have reported higher mean scores on the immature responses scale—including mechanisms of passive aggression, isolation, devaluation, and rationalization—compared to normative values [33]. Additionally, they have acknowledged lower mean scores on the neurotic and mature response scales, the latter involving mainly sublimation and humor.

Autonomy in the donation process is still a matter of debate. Recently, Weightman et al. underlined the risk of excessive paternalism in the donor selection process in the Australian context [11]. According to the authors, the current approach could be seen as too conservative, particularly when concerning medical selection criteria, which would overemphasize the risks for the donor without providing appropriate relevance to the benefits of donation for the recipients and, in many cases, the donor themselves. According to the existing literature, potential benefits for donors include improved perceived health and well-being—mainly as a result of the changes in lifestyle and awareness about physical health following donation—together with time, financial, and interpersonal benefits [19]. In this context, current practices may provide insufficient relevance to the individual risk tolerance of the candidate donors, as well as to their possibility of obtaining significant benefits, thus failing to respect their autonomy [11]. This position recalls recent studies that have suggested the expansion of the traditional donor evaluation and selection framework, based on acceptable risk levels, to a more nuanced and balanced individual donor risk-to-benefit ratio, which would include the assessment of potential tangible benefits [19].

Taken together, these different positions underline the need for a tailored, respectful approach to understanding the unique relational and psychosocial context of the donor-recipient dyad in order to ensure respect for autonomy and the best care for both the donor and the recipient.

#### 3.1.1. Donor Feelings and Motivations

A careful understanding of a donor’s feelings and motivations represents a relevant target of the psychosocial evaluation from a clinical and ethical perspective. Early research in the field has suggested that the decision to donate is settled before any medical information is provided to the potential donor [34]. This has to be kept in mind when assessing autonomy. Mazaris et al. summarized the main feelings and motivations of donors and recipients [13]. The main reason to donate would be the desire to help, as a natural response to another person’s suffering, together with a sense of moral duty [13]. Interestingly, in a qualitative study involving 20 subjects who donated a kidney to first-degree relatives and subsequently developed ESKD themselves [27], the participants did not frame their fulfillment of perceived obligations as a result of duty but rather as the result of volition and the consequence of love. Study participants explicitly denied feelings of pressure, describing donation as a consequence of the ordinary intrafamilial relationships involving donors and recipients rather than a consequence of their own extraordinary personality. Donors also frequently reported a sense of reciprocity if the roles were reversed, as well as the hope to ameliorate the whole family’s quality of life, which would otherwise be hampered by the limitations imposed by their kin’s diseases [19]. Less frequent motivations included religious beliefs or an explicit feeling of guilt. Most religions, including Catholicism, Judaism, and Buddhism, accept and support transplantations and organ donations [35].

Other psychosocial factors, such as gender and ethnicity, should be taken into account. For instance, one of the first surveys investigating donor motivations reported that men tend to perceive their gift as an extraordinary act and are more ambivalent, while women described donation as an extension of family obligation [36]. Approximately, 1700 children per year with ESKD undergo kidney transplantation in Europe and the United States of America, with a range of 30% to 50% of LDKT. According to a Collaborative Transplant Study report, maternal versus paternal living kidney transplant donation is associated with a lower rejection in young pediatric recipients: these data once more support the complexity of clinical and psychosocial factors entangled in the decision to donate, as well as in the family choice of the donor [37].

Notably, over the last few years, there has been an increase in cultural and ethnic diversity within Europe, including Italy, with a growing proportion of non-Italian native-speaker donors. In these cases, the employment of independent translators is mandatory, although it may lead to a non-fully verifiable communication of medical information. This fact could, in turn, limit the possibility of guaranteeing the interests and rights of the candidate donor and increasing the risk of coercion or, at least, of incomplete understanding [14].

#### 3.1.2. Recipient Feelings and Motivations

Surprisingly, relatively few studies have examined the unique perspective of individuals who have received a kidney from a living donor. In a qualitative study by Ummel et al., recipients provided a multifaceted and rich description of emotional reactions, including refusal, surprise, ambivalence, and guilt in the pre-transplant phase [38]. In the same study, all of the participants described the transplantation as a positive and life-changing experience, a “gift” that conferred them freedom and a new lease on life.

Recipient feelings of guilt, especially in the case of close relationships, have been previously documented [39,40]. In a retrospective study by Lee et al. (2020) involving 53 donor–recipient dyads, the recipients reported higher levels of depression and somatic concerns compared to donors in the pre-transplant phase [41]. Of interest was that the research showed that adolescents receiving parental grafts experienced strong feelings of obligation and indebtedness to the donor, which led to psychological distress, possibly derived from the parent-child conflict enhanced by the transplant experience [40]. More recently, a scoping review exploring the psychosocial needs of adolescent and young adult kidney transplant recipients identified several psychosocial unmet issues, including the need for emotional support, acceptance, direction, and equality in healthcare [42].

Clinical experience and research suggest that the decision to accept a kidney from a living donor may be sometimes difficult itself. A recent qualitative analysis explored the reasons for patients choosing dialysis or refusing kidney transplantation as renal replacement therapy [43,44]. The reasons included the perception of kidney transplantation as a renal replacement therapy without positive outcomes and the refusal to have an allograft. In the case of LDKT, patients were motivated in their refusal to accept living donation due to concerns about the donor’s health and the fear that the procedure would compromise their relationship with the donor [45].

### 3.2. Beneficence and Non-Maleficence

Beneficence is the second key principle of biomedical ethics. It is defined as the responsibility of the physician to ensure well-being by only acting in ways that will benefit patients and defend their rights. Non-maleficence refers to the principle of a physician’s obligation to not cause any harm. The practical application of non-maleficence is for the physician to weigh the benefits against the burdens of all interventions and treatments, to eschew those that are inappropriately burdensome, and to choose the best course of action for the patient [26]. These two principles are clearly related in logical and practical terms and will therefore be discussed together in this review. In LDKT, it is important to consider both the benefits and challenges that may come with the transplant, for both the recipient and the donor. As for the recipients, LDKT is associated with superior outcomes in terms of both graft and patient survival in comparison to transplantation from a deceased donor. According to the National Transplant Center (CNT) in Italy, the survival rates for patients undergoing a transplant from a deceased kidney donor are 97.3% and 92.1% after 1 and 5 years, respectively, compared to 98.8% and 96.9% in the case of living donation [46]. A kidney from a living donor transplant lasts on average 20 to 25 years, while a kidney from a deceased donor transplant lasts around 15 to 20 years [47]. Basiri et al. also found fewer medical complications in LDKT compared to deceased donor transplants [48]. The medical and psychosocial benefits of LDKT for recipients are therefore significant in terms of improved health and quality of life.

Nevertheless, it is important to point out that patients can face many challenges while on the waitlist for a kidney transplant. Indeed, they may report feelings of isolation, anxiety, and depression [49]. LDKT transplants generally reduce the waiting time [48] and, in the case of preemptive transplantation, permit one to avoid dialysis and its related limitations [50]. In a qualitative paper, Antoun et al. reported that, after receiving a kidney transplant, patients felt more independent and free, had an improved quality of life, and were able to do physical activity compared to before getting sick [51]. However, they also reported anxiety and fear that the transplanted kidney might fail. Additionally, after the transplant, patients may feel a sense of guilt for receiving the organ of someone who died or because others they knew in dialysis did not receive one, or they may have a fear of getting diseases from the new organ or could develop a feeling of isolation as they stop going to the dialysis unit [44].

With regard to beneficence and non-maleficence, it is also important to note that, after a kidney transplant, recipients have to take immunosuppressants, which may result in severe side effects. They also have to be isolated for a certain period of time in order to prevent infections. Patients who have undergone a transplant suffering from psychological distress may have difficulties in adhering to immunosuppressive therapy. This could, in turn, increase the risk of organ rejection. Sleep disorders are also common post-transplant, although less frequent than during dialysis. The same applies for cognitive deterioration. Therefore, it is very important for transplanted patients to undergo psychological or psychiatric follow-ups [21].

Concerning the donor, the current evaluation and selection model is based on the acceptable risk paradigm. In the absence of direct benefits in terms of physical health, risks must be limited and controlled. At present, the safety of kidney donation has been extensively documented, with a very low risk of major harm, e.g., kidney failure (0.9%) or death (0.03%) [52,53]. Long-term physical and psychological morbidity is limited with no impact on life span [54,55]. However, clinical data have shown that donors are at increased risk of hypertension, preeclampsia, and ESKD [56,57].

As for psychosocial outcomes, research has reported no significant changes or even an improvement in the quality of life of living donors one year after the donation [55,58]. Interpersonal relationships appear to remain the same or improve after donation [54], and the majority of donors did not regret their decision, even in the case of an adverse outcome for the recipient or the donor themselves [27,59].

More recently, a study conducted by Van Pilsum Rasmussen et al. qualitatively explored the experience of donors, dividing potential benefits of donation into different areas, i.e., interpersonal, wellness and health, time, and financial benefits [19]. For example, the donors reported increased insight into their own health, a cessation of smoking, and reduced stress. They also reported a decrease in caregiving burden and an improvement in their financial situation after going back to work. Additionally, if they donated to a relative, they felt closer to them and felt they avoided the guilt of not donating.

A minority of donors reported negative psychosocial outcomes, such as post-donation anxiety, depression, and regret in being living kidney donors. Post-donation depressive or anxiety symptoms have been reported in 5–23% and 6–14% of donors, respectively. Concerns included living with one kidney, complications of nephrectomy, insults to one’s own health, future kidney problems, financial issues, and recipient outcomes. [54]. A more recent cross-sectional study by Holscher et al. investigated anxiety and depression using the Generalized Anxiety Disorder 2-item (GAD-2) and the Patient Health Questionnaire 2-item (PHQ-2) scales in a sample of 825 living kidney donors at a median since donation time of 6 years [59]. Overall, 5.5% of the study participants screened positive for anxiety, 4.2% for depression, and 2.1% reported regretting their donation. Medical complications experienced by the recipient or by the donor have been shown to predict a worsening of psychological symptoms one year after kidney donation [60]. Taken together, these data support the importance of transplant professionals in screening, evaluating, and providing aftercare for living organ donors [29].

### 3.3. Justice

As an ethical principle, justice implies two main aspects: the personal aspect is the appropriate, fair, and equitable treatment of a patient; and the broader aspect is the appropriate distribution of medical resources [26]. There is a great disparity in the access to transplants between high- and low-income countries. Elrggal et al. reported that, in low-income countries, many patients do not receive effective treatment (96%) compared to high-income countries (40%), and they usually have access to live donor kidney transplants only [61]. Barriers to transplantation may include cultural factors, misconceptions regarding the transplant (attributable to a lack of education on the topic), a fear of dying earlier if they donate, a distrust in the doctors, an inadequate understanding of the concept of brain death, or false myths. There is a particularly negative connotation for deceased donations in Muslim countries. Furthermore, the governments do not provide sufficient financial support for donations in low-income countries. Additionally, it has been reported that, in low-income countries, there is an even larger gap between supply and demand, and many transplants come from organ trafficking [61].

In this respect, a study in 2007 found that organ trafficking (for example, taking organs from executed prisoners) often occurs due to the lack of access to organs for transplants. This practice is associated with many ethical and medical issues. For example, the lack of medical tests run on the organs results in a greater transmission of infections, such as HIV and hepatitis [62]. To address this issue, the Declaration of Istanbul was released in 2008, and was updated in 2018, to prevent transplant tourism, organ trafficking, and to create ethical programs for organ donation/transplants [63].

Depending on the religion, country, or even region, people may have different views regarding organ donations and transplantation. For example, Jehovah’s Witnesses do not accept allogeneic transfusions but allow organ transplants. This raises some ethical questions and debates when the organ is not directly donated to them but rather comes from a deceased donor. Cummins and Nicoli explained that people may find it unfair that Jehovah’s Witnesses may receive an organ rather than someone who fully accepts the standards of care (e.g., because they do not accept transfusions during transplant procedures). However, it would be unethical to exclude them from the transplant lists as the outcomes of a transplant without transfusions are comparable to those of a transplant with transfusions [64].

In countries where transplantations are routinely performed, barriers to access still exist for ethnic minorities. For instance, a scoping review exploring inequities in access to living donor kidney transplantation in Canada reported how East Asian, South Asian, African Caribbean and Black Canadian communities face barriers in accessing culturally appropriate medical knowledge and care, leading to inequitable access to kidney transplants. Additional barriers may include religious and spiritual concerns, stigma toward ESKD and kidney transplants, health beliefs, social determinants of health, and experiences of systemic racism in healthcare [65].

During the COVID-19 pandemic, new bioethical and public health questions arose regarding kidney transplantations. In fact, there has been a sharp increase in mortality for patients on the waitlist for kidney transplants. From a bioethical point of view, one aspect considered was the importance of keeping the patients safe, avoiding unnecessary accesses to hospitals, and exposure to potential sources of infection. Another relevant point discussed was the need to provide recommendations on SARS-CoV-2 vaccines for both patients (if they should be mandatory and whether they should be taken before the transplant) and workers, as well as even whether the transplant-related activity should continue or be stopped. Additionally, ethical questions arose when it came to deciding how to distribute the scarce medical resources available as kidney donations and centers doing living donor transplantations decreased during the pandemic [24].

### 3.4. Kidney Donation from a Living Donor to a Stranger (Unspecified Kidney Donation, Altruistic Donation, and Samaritan Donation)

Unspecified kidney donation (UKD), or Samaritan donation, refers to the living donation of a kidney to a stranger. The specificity of this option from an ethical and psychosocial point of view is that the donor and the recipient do not have any family or emotional bond, i.e., they do not know each other. UKD implies a complete extraneousness—either biological or psychological—between the donor and the recipient in the absence of any kind of reward. The donation process is covered by strict anonymity in Italy [66]. Unspecified kidney donors may trigger a chain of transplants—altruistic donor chains—between two or more incompatible pairs in domino-paired kidney exchange programs or through donating to a patient on a deceased donor waiting list [67]. Introduced in the early 2000s, UKD raised intense ethical and clinical debate, although it may represent a precious resource for enhancing LDKT programs.

According to the Italian National Bioethics Committee (INBC), the Samaritan donation represents a supererogatory act; as such, it is ethically significant for the solidarity motivations inspiring it. The INBC underlines that kidney donation to a stranger does not involve higher medical risks for the living donor than those implied in other forms of ex vivo kidney removal. According to Italian national legislation, gratuitous organ donation must be carried out in organ transplant centers, university institutes, and hospitals involved in scientific research [66]. The first ten cases of UKD are currently under the supervision of a dedicated national program coordinated by the National Transplant Center (CNT).

A major concern with this method regards the uncertainty about the donor’s motivation, and, in particular, whether the desire to donate to a stranger may be a symptom of psychopathology. While increased clinical experience has partially attenuated this preoccupation, the concern has not been completely eliminated [68]. Analogously, the American guidelines for the Psychosocial Evaluation of Living Unrelated Kidney Donors recommend a careful psychosocial evaluation process for UKD [69]. Kranenburg et al. provided a review of the literature describing the heterogeneity of the psychosocial evaluation practices in UKD based on clinical interviews and additional psychometric tests. The two major reasons for donor exclusion reported at the time were current psychopathology—mainly including severe depression or psychological instability—and motivational issues, including unrealistic expectations, desire for media attention, or monetary compensation [70].

Dew et al. highlighted potential risk factors for poor subsequent donors’ psychosocial outcomes, including ambivalence; motives reflecting a desire for recognition; a desire to use the donation to develop a personal relationship; multiple family stressors/obligations/concerns; subordinate relationship or other evidence of coercion; and evidence of, or expectation of, secondary gain (e.g., avoidance of military duty, financial support from the recipient, etc. [69]). A careful and deep understanding of the candidate donor’s motivation is therefore imperative to guarantee the donation process as the expression of an autonomous decision.

Concerning beneficence and non-maleficence, psychological benefits are generally seen as a major moral justification for the surgical practice of living donor nephrectomy [28]. In the case of UKD, the donor’s potential benefits are not clear compared to specified kidney donation, where the potential harm to the donor due to the perceived suffering of the recipient can be more understandable. Previous research has found that UKD is primarily motivated by a desire to help: the desire/drive to donate is frequently concomitant with similar altruistic behaviors [67,68].

The finding of a retrospective study by Massey et al. (2022) involving 114 unspecified kidney donors may shed some light on this issue. At an average follow-up of 7 years after donation, participants reported a significantly higher positive mental health score than the Dutch general population on emotional and social well-being. Participants were found to experience positive emotions and a sense of being involved and having contributed to society. With regard to psychological symptoms, the aforementioned study did not find evidence of significant changes between the pre-donation and post-donation periods [67]. Similarly, in the study by Bramsted et al. (2008) investigating the long-term follow-up of a sample of 13 Samaritan donors, ten kidney donors reported overall positive outcomes in terms of perceived physical health, quality of life, and personal experience. The survey was integrated by the participants’ narrative comments about individual adverse events which, even if rare, were not captured by the questionnaires but deserved consideration by the transplant team. The authors underlined the need for follow-up visits addressing psychosocial issues if present and providing appropriate support [71].

Kurleto et al. (2020) investigated the motivations, characteristics, and perioperative experiences of 115 non-related living donors recruited by the Matnat Chaim organization in Israel. Matnat Chaim is a non-profit organization promoting non-directed living kidney donation. The majority of study participants reported a willingness to help and a desire to do good as their main motivations to donate. The majority of donors (78.3%) reported stable health status after donation; however, 16.5% experienced clinical problems (e.g., wound infection, more pain than expected, etc.) and 5.2% experienced psychological complications. Of interest was that 18% of donors described their perceived health as improved after the donation [72].

Of note, there are enormous epidemiological variations across different countries in which UKD is allowed that are still poorly understood. According to the data reported in previous studies on this topic, there were 184 kidney altruistic donations in the United States in 2014 [71] compared to 89 in the UK between 2017 and 2018 [68] and 8 Samaritan donations performed in Italy between 2015 and 2019, and the latter led to 26 transplants [73].

## 4. Discussion

LDKT currently represents an established procedure. Nonetheless, this topic is still the subject of an intense ethical debate; one that is mainly centered on the issue of performing a major surgical procedure on healthy individuals for the benefit of someone else. On the one hand, epidemiological and clinical research has provided initial data on donor safety, reporting limited and controlled risks and potential emotional and psychosocial tangible benefits. On the other hand, the implementation of LDKT programs, together with immunological, pharmacological, and surgical advances, has led to a continuously evolving scenario. For instance, in the case of paired exchange programs—which are developed to achieve transplant between incompatible pairs—there are now more advanced strategies, with increasing complexity, to maximize the number of potential beneficiaries. These programs may involve a non-directed altruistic donor to initiate a large donation chain or a kidney selection from the pool of deceased donors to initiate the chain (i.e., such as in the case of the DECeased-Kidney-paired-exchange [DECK]) [74]. All these options raise ethical concerns regarding the protection of live donors in terms of their authentic autonomy, understanding, and beneficence/non-maleficence ratio.

A careful understanding of the psychosocial outcomes, including donor reaction to the donation experience and quality of life, is crucial not only from a clinical but also from an ethical point of view as donor motivation and potential benefits are mainly based on these aspects [28].

The emotional and psychosocial benefits of living organ donation have, largely, been documented. Living kidney donors may expect improvements in personal growth, interpersonal relationships (particularly their relationship with the recipient), self-esteem, social engagement, and spiritual life [38,54,75]. As previously discussed, a qualitative study by Van Pilsum Rasmussen described a spectrum of potential tangible and measurable benefits experienced by donors to be incorporated into the traditional evaluation model based on acceptable levels of risk [19]. Weightman et al. (2022) underlined the need to respect donor autonomy against the risk of excessive paternalism and protection [11].

Overall, living kidney donors report positive feelings about the donation experience and satisfactory levels of quality of life. However, as observed by Dew (2017), data assessed using quantitative standardized scales may be insufficient to capture the risk in individual donors [18]. Qualitative studies provide a more nuanced perspective including positive feelings together with vulnerability, anxiety, and ambivalence. Some donors have depicted the donation process as an “overwhelming experience” that was not always positive and often marked by a feeling of vulnerability after surgery [20]. The relationship with the recipient was closer after the donation but it was necessary to undergo a renegotiation of roles and expectations. LDKT represents a very demanding and particularly stressful event with profound bio-psychosocial implications for the recipient, the donor, and often the entire family context. Major surgery may threaten the sense of continuity and personal integrity, causing strong emotions in the perioperative period. This process of adaptation may invest both the recipient (in terms of integrating the new organ) and the donor (in terms of loss of a part of themselves) [21].

Psychosocial evaluations take place in the pre-donation period, which is often very distressing for donors [20,38]. Concerning autonomy, professionals must be aware of the conscious and unconscious strategies that are potentially adopted by donors to influence the selection process of the transplant team during the pre-donation period. Moreover, during the evaluation period and the follow-up of LDKT, the literature has extensively documented the existence of a social desirability bias, which is the tendency of individuals to provide socially desirable answers when responding to personality tests or surveys [20].

Although donors often describe their decision as obvious, they may face dilemmas, ambivalence, and anxiety. Professionals should be aware of this multifaceted situation from an ethical and clinical point of view. Even in the case of a strict kinship relationship, one could argue to what extent the recipient and donor’s autonomy is complete, especially in the case of a minor or fragile recipient.

Ethics and the assessment of autonomy profoundly deal with the respect—and protection—of the most fragile individuals in clinical contexts. In the case of LDKT, the roles—i.e., who is the most fragile, if any—may not always be obvious in the donor–recipient dyad. Moreover, a rational choice, based on the clarity and completeness of the information received, may not necessarily coincide with a free choice. In LDKT, the psychosocial evaluation stands at the crossroads of all these issues. The psychosocial evaluation process is often perceived as an additional demanding part of the already complex, long, and uncertain medical assessments that are undertaken by patients and families (and sometimes by the transplant team itself) [76]. Nevertheless, it may represent an important opportunity to capture unmet needs, worries, and difficulties, as well as may help in building a supportive network.

Interestingly but quite surprisingly, the psychosocial assessment process is still largely heterogeneous among countries and even transplant centers in terms of the professionals involved (e.g., nurses, social workers, psychiatrists, and psychologists) and assessment instruments. Previous studies have emphasized the need for shared, standardized procedures while respecting cultural, social, and value diversity [29].

Even in the post-transplant period, routine follow-up assessments addressing psychosocial issues should be implemented. Of note is that some donors have reported feeling forgotten, lost, and abandoned after donation, whereas they were considered “sensational” before donation [20].

## 5. Conclusions

To our knowledge, this is the first review that has comprehensively summarized the ethical issues in living kidney transplantation from a psychosocial point of view and has included articles reporting data on both donors and recipients. However, our paper should be discussed in the light of some limitations. First, we did not perform a systematic search in more databases following the PRISMA guidelines [77]. Also, we did not evaluate the quality of the included studies. The theoretical background was designed to be conformant with Italian regulations on this topic, which are consistent with the majority of European countries but may not be generalizable to other contexts. Further research is needed to provide a more in-depth insight and sensitive understanding of the ethical issues of LDKT across the world. However, given the heterogeneity of the literature on the topic, we believe that the present narrative review may be sufficiently comprehensive to represent a first step in exploring the ethical issues of LDKT from a psychosocial perspective. It must also be acknowledged that more studies are needed to fully understand the utility and limitations of different organ origins and surgical procedures, with the final aim being to identify what works better and for whom.

Taken together, the findings of our review support the importance of reflecting on psychosocial aspects to better understand the unique experience of the donor-recipient dyad, or of the single donor in the case of an altruistic donation, making the transplant an expression of autonomy, beneficence, and justice.

## Data Availability

Not applicable.
